# One‐Minute Preparation of Iron Foam‐Drug Implant for Ultralow‐Power Magnetic Hyperthermia‐Based Combination Therapy of Tumors in Vivo

**DOI:** 10.1002/advs.202307823

**Published:** 2024-01-02

**Authors:** Guangchao Xie, Bingjie Li, Xuejun Zhang, Jiaojiao Yu, Shao‐Kai Sun

**Affiliations:** ^1^ Department of Diagnostic and Therapeutic Ultrasonography Tianjin Medical University Cancer Institute and Hospital National Clinical Research Center of Cancer Key Laboratory of Cancer Prevention and Therapy Tianjin 300060 China; ^2^ School of Medical Imaging Tianjin Medical University Tianjin 300203 China; ^3^ Department of Radiology and Tianjin Key Laboratory of Functional Imaging Tianjin Medical University General Hospital Tianjin 300052 China

**Keywords:** combination treatment, facile drug loading, iron foam, low‐power magnetic hyperthermia

## Abstract

The magnetic hyperthermia‐based combination therapy (MHCT) is a powerful tumor treatment approach due to its unlimited tissue penetration depth and synergistic therapeutic effect. However, strong magnetic hyperthermia and facile drug loading are incompatible with current MHCT platforms. Herein, an iron foam (IF)‐drug implant is established in an ultra‐facile and universal way for ultralow‐power MHCT of tumors in vivo for the first time. The IF‐drug implant is fabricated by simply immersing IF in a drug solution at an adjustable concentration for 1 min. Continuous metal structure of IF enables ultra‐high efficient magnetic hyperthermia based on eddy current thermal effect, and its porous feature provides great space for loading various hydrophilic and hydrophobic drugs via “capillary action”. In addition, the IF has the merits of low cost, customizable size and shape, and good biocompatibility and biodegradability, benefiting reproducible and large‐scale preparation of IF‐drug implants for biological application. As a proof of concept, IF‐doxorubicin (IF‐DOX) is used for combined tumor treatment in vivo and achieves excellent therapeutic efficacy at a magnetic field intensity an order of magnitude lower than the threshold for biosafety application. The proposed IF‐drug implant provides a handy and universal method for the fabrication of MHCT platforms for ultralow‐power combination therapy.

## Graphical abstract

An iron foam (IF)‐drug implant is established in an ultra‐facile way for ultralow‐power magnetic hyperthermia‐based combination therapy (MHCT) of tumors in vivo for the first time, which solves the incompatible problem of high‐efficient magnetic hyperthermia and ultra‐simple and fast drug loading, and lays down a convenient and universal MHCT platform for tumor therapy in vivo.

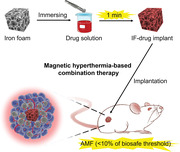

## 1 Introduction

Magnetic hyperthermia therapy (MHT) is a promising thermal therapy method for tumors, which has the merits of low invasiveness, minimal side effects, and excellent tissue penetration, and has realized great progress in fundamental research and clinical trials.^[^ ^1^ ^]^ However, the high temperature is essential for a single MHT to eradicate tumors, which increases the risk of potential damage to adjacent tissue.^[^ ^2^ ^]^ In addition, the therapeutic effect of a single MHT is not ideal for whole tumor tissue due to its inhomogeneous distribution of temperature.^[^ ^3^ ^]^ To solve these problems, tremendous efforts have been devoted to magnetic hyperthermia‐based combination therapy (MHCT) by combining MHT with chemotherapy, immunotherapy, and others, which has been regarded as a promising synergistic therapeutic strategy.^[^ ^4^ ^]^ The MHCT not only facilitates complete tumor eradication at lower magnetic field intensities and smaller drug doses, but also minimizes damage to surrounding healthy tissues, thereby yielding a highly effective treatment outcome while mitigating side effects.^[^ ^1^, ^5^ ^]^

The current MHCT platforms can be divided into two categories: magnetic nanoparticles (NPs) and liquid/solid metal implant‐based platforms.^[^ ^1^, ^5^, ^6^ ^]^ For the former, magnetic NPs (e.g., Fe_3_O_4_ NPs, Fe_2_O_3_ NPs, and Fe NPs) were synthesized with adjustable morphology and size, and their high specific surface area enabled highly efficient drug loading.^[^ ^7^ ^]^ However, despite the elaborated design, tedious synthesis process, harsh reaction conditions, and complex post‐modifications, the magnetic NPs, which generate magnetic hyperthermia based on relaxation loss and hysteresis loss mechanisms, can hardly own high magnetothermal capability.^[^ ^8^ ^]^ As a result, repeated administrations and high‐power alternating magnetic fields (AMF) are required in magnetic NPs‐based MHCT, severely hindering its biological application. In contrast, metal implants with large size (millimeter‐scale) and continuous metal structures can generate huge heat through eddy current thermal effect even in low‐power AMF, thus exhibiting powerful magnetothermal capability.^[^ ^6^, ^9^ ^]^ However, metal implants with dense structures and small specific surface areas often require further complex corrosion or electroplating strategies to achieve a limited drug loading, which greatly increases the difficulty and reduces the efficiency of drug loading. Therefore, it is urgent to develop a universal MHCT platform with superior magnetothermal properties and convenient and efficient drug‐loading capability for tumor treatment.

Iron foam (IF) is a zero‐valent iron block with a uniform porous structure and is widely used in the fields of tail gas purification, battery electrodes, electromagnetic wave shielding, and biological scaffolds.^[^ ^10^ ^]^ Continuous metal structure of IF enables highly efficient magnetic hyperthermia like solid metal based on eddy current thermal effect. Moreover, its abundant pore structure provides a huge space for drug loading in a simple adsorption method. Furthermore, it has the advantages of low cost, customizable size and shape, and superior scale production capability. Additionally, iron performs many important physiological functions in the human body as an essential trace element, ensuring the biosafety of IF for application in vivo.^[^ ^11^ ^]^ Besides, implant therapy is an indispensable treatment method in clinics for tumor patients who must preserve vital functional tissues, lose the opportunity or value of surgery, and refuse radical surgery.^[^ ^12^ ^]^ Therefore, IF with a porous structure is expected to be an excellent substitute for magnetic NPs and solid metal implants for MHCT.

**Scheme 1:**
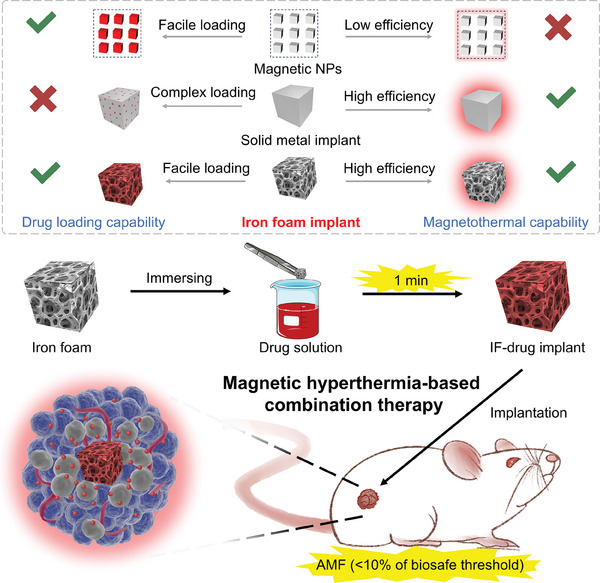
Schematic illustration of 1‐min preparation of iron foam‐drug implant for ultralow‐power MHCT of tumors in vivo. Upper panel: comparison of different magnetic hyperthermia materials in terms of drug loading capability and magnetic hyperthermia capability. Magnetic nanoparticles: facile drug loading process (√), low magnetothermal heating efficiency (×). Solid metal implant: complex drug loading process (×), high magnetothermal heating efficiency (√). Iron foam implant: facile drug loading process (√), high magnetothermal heating efficiency (√).

Herein, the IF‐drug implant was fabricated as a universal platform for ultralow‐power MHCT of tumors in vivo (Scheme 1). The implant was synthesized by immersing commercial IF in the drug solution based on the “capillary action”, and this preparation process can be finished in 1 min without the need for any special equipment and conditions. The ultra‐fast and straightforward drug loading process, 1‐min preparation, significantly reduces energy and time consumption, enabling this method to be used in a ready‐to‐use manner. The IF can be employed to load various drugs including hydrophilic and hydrophobic cargoes high efficiently, and drug loading had a neglectable influence on magnetothermal capacity. Moreover, IF has the advantages of low cost, customizable size and shape, superior scale production capability, benefiting reproducible and facile fabrication of various IF‐drug implants. As a proof of concept, the clinical chemotherapeutic drug of doxorubicin (DOX) was selected to construct IF‐DOX implant for in vivo application. The IF‐DOX implant achieved efficient combined magnetic hyperthermia and chemotherapy efficacy of tumors in vivo under an ultralow‐power magnetic field intensity (H_appl_·f_appl_ = 3.78 × 10^8^ A m^−1^ s^−1^) successfully, which is an order of magnitude lower than the threshold for biosafety application (H_appl_·f_appl_ = 5 × 10^9^ A m^−1^ s^−1^).^[^ ^13^ ^]^ As far as we know, this is the first time that the unification of exceptionally high magnetic hyperthermia capacity and ultra‐facile drug loading has been achieved.

## 2 Results and Discussion

### 2.1 Preparation and Characterization of IF‐Drug Implant

**Figure 1:**
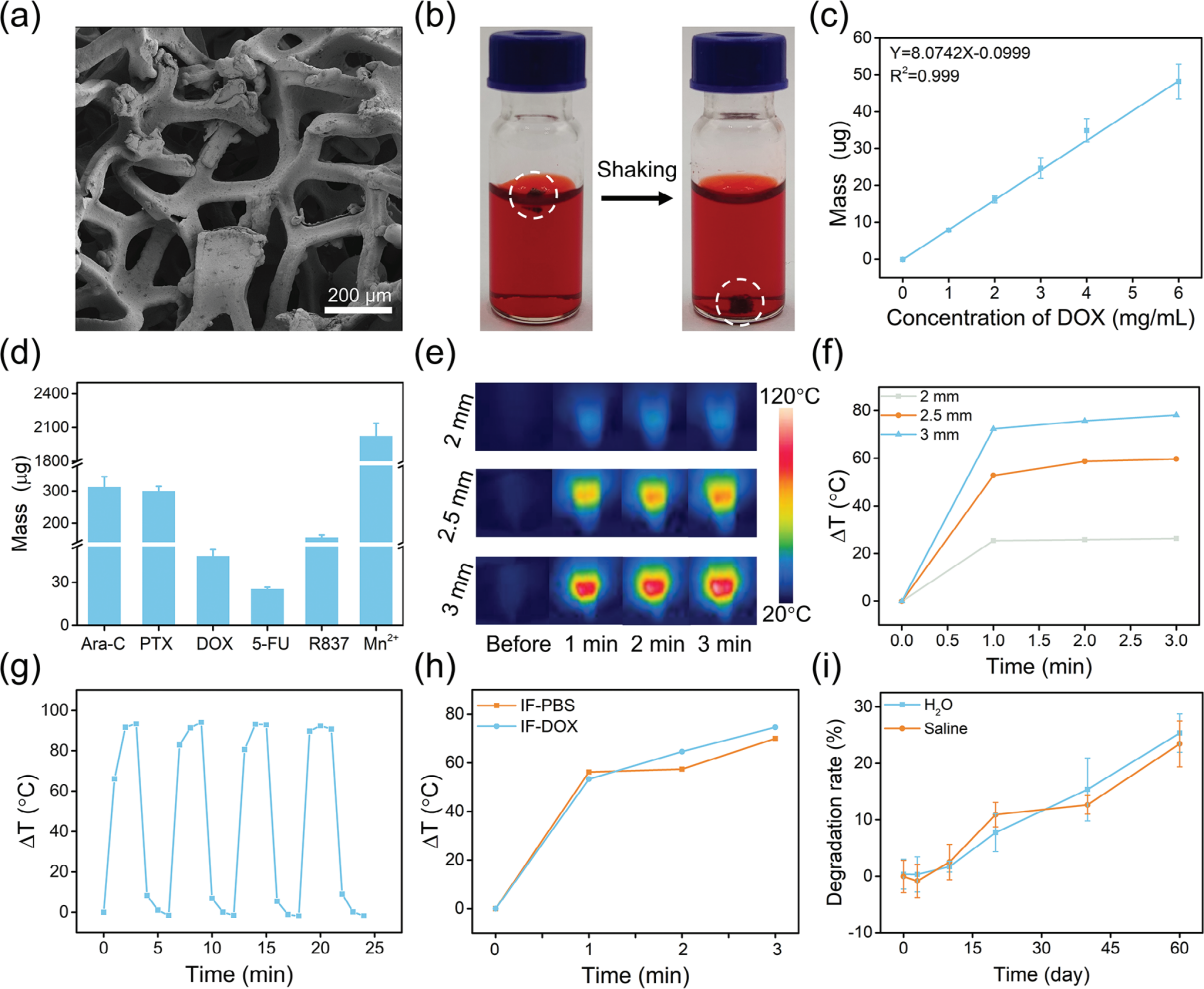
a) SEM image of IF. b) Photos of IF (2 × 2 × 2 mm) in DOX solution before and after shaking. c) Relationship between DOX loading mass of IF and DOX solution concentration. d) Loading mass of IF for different drugs. e) Thermal images of IF with different sizes in AMF at different times. f) Heating curves of IF with different sizes in AMF at different times. g) Heating and cooling curves of IF (2 × 2 × 2 mm) in AMF for 4 cycles. h) Heating curves of IF‐PBS (2 × 2 × 2 mm) and IF‐DOX (2 × 2 × 2 mm) in AMF. i) Degradation rate of IF (2 × 2 × 2 mm) in H_2_O and saline.

Commercial IF has the merits of low cost, reproducible preparation, superior large‐scale production capability, and can be commercially customized according to the needs of the composition, size, and shape. As shown in Figure S1 (Supporting Information), three kinds of IF cubes with different sizes were customized in this experiment (2 × 2 × 2 mm, 2.5 × 2.5 × 2.5 mm, and 3 × 3 × 3 mm), and the porosity was as high as 92.5%. The scanning electron microscope (SEM) images of IF showed a continuous network‐like structure with micrometer‐level pores, which provides a huge space for loading cargo (**Figure** **1**a). Due to the continuous metal structure, the IF has a good electrical conductivity of ≈2.11 х 10^5^ S m^−1^ and can act as a good connector in the circuit (Figure S2, Supporting Information), endowing the generation of huge heat in an AMF through eddy thermal effect. The IF can float on the surface after putting it into liquid (DOX:6 mg mL^−1^ and water) due to its porous structure (Figure 1b; Figure S3, Supporting Information), and they sink quickly as liquid enters the internal pores of IF slightly shaking. As IF possesses pores on the scale of several hundred micrometers, its mechanism for drug loading relies on the capillary action of these pores with the solution. This is in contrast to the drug adsorption process in porous materials with nanosized pores, which typically involves various physical and chemical interactions between the porous materials and drug molecules. As shown in Figure S4 (Supporting Information), the concentration of the drug solution remained unaltered pre‐ and post‐loading, thereby confirming that IF facilitates drug loading through simple capillary action. The liquid absorbed by IF can be stably stored in the internal pores, and will not flow out casually (Figure S5, Supporting Information). This phenomenon indicated IF was capable of absorbing the surrounding liquid in a short time based on the capillary action just like a sponge absorbs water.

The kinetics of liquid loading of IF (2 × 2 × 2 mm) was investigated to understand the kinetics involved in loading various cargo solutions into IF by the mass difference method. Figure S6 (Supporting Information) shows that IF can quickly adsorb the liquid and reach a stable state by immersing IF in water for 30 s, which indicates IF has great potential in loading any drugs dissolved in liquid. To investigate drug loading efficiency, different concentrations of DOX solutions were used to synthesize IF‐DOX implants, and the amounts of loaded DOX were quantified by UV–vis absorption. As shown in Figure 1c, the DOX loading mass in IF‐DOX implant increased linearly with the enhancement of DOX solution concentration. When the concentration of DOX solution was 6 mg mL^−1^ (approach to its solubility), one IF can load ≈48.21 µg of DOX. Besides the hydrophilic drugs like DOX, there are many hydrophobic drugs in the clinic, and they can be easily loaded in IF by dissolving them in individually biosafe and benign solvents (PBS, dilute NaOH, water, ethanol, and oleic acid) in the same manner. Totally, we studied the loading ability of IF toward various drugs including five chemotherapeutic drugs and two immunoregulator drugs. As shown in Figure 1d, one IF (2 × 2 × 2 mm) can load ≈313.12 µg Ara‐C, 300.11 µg PTX, 25.37 µg 5‐FU, 154.77 µg R837, and 2.02 mg Mn^2+^, respectively. It is worth noting that solvents such as PBS (pH 6), dilute NaOH (pH 8.5), H_2_O, and oleic acid have good biosafety, and the constructed IF‐drug can be directly transplanted into living organisms. When the solvent was ethanol, the constructed IF‐drug implant can be used after drying at room temperature. In addition, Figure S7 (Supporting Information) demonstrates that different IFs (2 × 2 × 2 mm) can load drugs with similar mass, with a relative standard deviation (RSD) of 1.98%, proving the excellent drug‐loading reproducibility of IFs. These results indicated IF can serve as a high‐performance and universal carrier to quantitatively load hydrophilic and hydrophobic drugs according to the actual needs by simply tuning the concentration of drug solution in a short time (1 min). The larger the solubility of the drug, the higher the loading capacity of IF for the drug, so a high drug loading capability can be achieved by dissolving the drugs in benign and biosafe media.

Unlike the drug‐loaded nanoparticles, which are intravenously injected into the body and then will flow into the bloodstream, the IF‐DOX implant will be enveloped closely by the tumor tissue after being implanted into the tumor site. Therefore, we investigate the release behavior of DOX in vitro by embedding the IF‐DOX implant into pork tissue. As shown in Figure S8 (Supporting Information), half of the loaded DOX was released from the IF‐DOX implant in the pork tissue in the first 10 min, and the majority of the remaining DOX was gradually released in 3 h. Compared to the burst release of DOX in IF‐DOX implant in solution, the IF‐DOX implant exhibited a controlled release manner due to the coating of surrounding tissues, which was beneficial for local treatment.

### 2.2 Magnetothermal Performance of IF

Different from the traditional magnetic NPs which mainly produce heat through relaxation loss and hysteresis loss, IF with a continuous metal structure generates heat mainly through an efficient eddy current loss mechanism in AMF.^[^ ^9c^ ^]^ First, magnetothermal heating performance of IF with different sizes was studied at an ultralow AMF (an order of magnitude lower than the threshold for biosafety application (H_appl_·f_appl_ = 5 × 10^9^ A m^−1^ s^−1^)).^[^ ^13^ ^]^ As shown in Figure 1e,f, the magnetic heating effect was obviously enhanced with the increase of IF size in an ultralow magnetic field intensity (H_appl_·f_appl_ = 1.44 × 10^8^ A m^−1^ s^−1^). The temperature of IF cubes with sides of 2, 2.5, and 3 mm was raised by 26.3, 59.7, and 78.1 °C within 3 min, respectively. In addition, with the increase of magnetic field intensity, the heating efficiency of IF was enhanced obviously. It is worth noting that even the smallest IF (2 × 2 × 2 mm) showed a remarkable temperature increase by 93 °C within 3 min under the condition of H_appl_·f_appl_ = 3.78 × 10^8^ A m^−1^ s^−1^ (Figure S9, Supporting Information). These results demonstrate that IF had an outstanding magnetic heating efficiency. Besides, the stability of the heating capacity was studied by repeated heating and cooling processes at an AMF (H_appl_·f_appl_ = 3.78 × 10^8^ A m^−1^ s^−1^). Figure 1g shows that there is no decline in elevated temperature during four cycles, which proves good magnetothermal heating stability of IF. Moreover, Figure S10 (Supporting Information) shows that these different IFs (2 × 2 × 2 mm) exhibited similar magnetothermal heating change under the same magnetic field condition, with an RSD of 3.16%, which indicated the IF had excellent reproducibility of magnetothermal heating. Furthermore, compared with CoFe_2_O_4_ nanoparticles, one of the best magnetic nanomaterials with good magnetocaloric effect reported in a previous study,^[^ ^1b^ ^]^ whether they were in powder state or immersed in H_2_O IF both showed significantly better magnetothermal heating ability at the same magnetic field condition (Figure S11, Supporting Information).

The unique advantage of MHT is the excellent tissue penetration depth.^[^ ^1d^ ^]^ The magnetothermal heating capability of IF in deep tissue was evaluated using fresh pig liver to reflect the temperature change. As shown in Figure S12 (Supporting Information), the color of the center at pig liver tissue with IF implant changed from dark red to white after being heated in AMF for 6 min, which indicated the MHT with IF was capable of penetrating tissue deeply. However, in the PBS‐injection group, there was no color change in the center of the pig liver after heating in AMF with the same power and time. In addition, the color of the thermochromic material around the IF changed from light pink to amaranth after heating in AMF, while free thermochromic material had no obvious color change (Figure S13, Supporting Information). These results proved that IF‐based MHT exhibited excellent tissue penetration ability.

Considering the large amount of liquid present in IF after the formation of the IF‐drug implant, we further evaluated the magnetothermal performance of IF‐PBS. The result indicated the IF‐PBS also exhibits an excellent magnetothermal capability, and the temperature evaluation can reach as high as 70 °C within 3 min under the condition of H_appl_·f_appl_ = 3.78 × 10^8^ A m^−1^ s^−1^ (Figure 1h). Moreover, the magnetothermal performance of IF‐DOX implant after loading DOX was nearly the same as that of IF‐PBS, indicating that loading drugs did not affect the heating of IF. These results proved that IF owned ultrahigh efficient heating efficiency, excellent tissue penetration ability, and good magnetothermal stability regardless of loading cargoes or not, and had great potential for further biological application.

### 2.3 Degradation of IF in Vitro

To investigate the degradation of IF in vitro, IF was incubated in water and saline. A large amount of flocculent rust was generated gradually indicating the degradation of IF during 60 days. Quantitative analysis results revealed that IF (2 × 2 × 2 mm) degraded by 25.36% and 23.45% in water and saline, respectively (Figure 1i). These results demonstrate that IF has good degradability in vitro.

### 2.4 Cytotoxicity of IF

Cytotoxicity of IF was evaluated using 4T1 cells by standard MTT assay. Small magnets were placed on the outer side of the wells to make IF stick to the inner side of the 96‐well plates to avoid mechanical compression damage caused by the weight of IF during the incubation process (Figure S14, Supporting Information). The cell viability of treated cells was over 81% after incubating with one IF (2 × 2 × 2 mm) for 24 h, which demonstrated the low cell toxicity of IF (Figure S15, Supporting Information).

### 2.5 Combination Therapy of Tumor Cells Using IF‐DOX Implant

**Figure 2:**
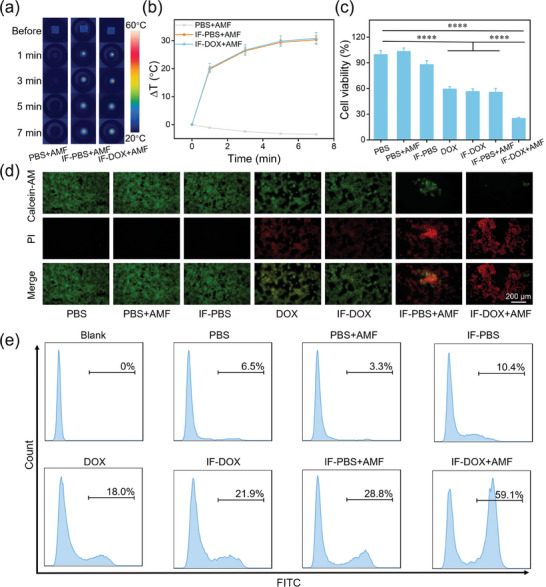
a) Thermal images of 4T1 cells with various operations. b) Heating curves of 4T1 cells with various operations. c) Cell viability of 4T1 cells after various operations. Statistical analysis was determined by one‐way ANOVA; ^****^ *p* < 0.0001. d) Fluorescence images of 4T1 cells after various operations. e) Cell apoptosis status of 4T1 cells analyzed by flow cytometry.

To investigate the therapeutic efficacy of IF‐DOX in vitro, the viabilities of 4T1 cells were determined after various treatments: i) PBS, ii) PBS+AMF, iii) IF‐PBS, iv) DOX, v) IF‐DOX, vi) IF‐PBS+AMF, vii) IF‐DOX+AMF. During the heating process in AMF, the temperature of the PBS+AMF group did not rise significantly, while the temperature of IF‐PBS+AMF and IF‐DOX+AMF groups increased by ≈30 °C in 7 min (**Figure** **2**a,b). These results revealed the combination of IF and AMF can lead to an obvious magnetothermal effect at the cellular level. The MTT assay results revealed that almost all the cells were alive in the PBS+AMF group, and 88% of cells survived in the IF‐PBS group (Figure 2c). However, there was significant cell death in the DOX, IF‐DOX, and IF‐PBS+AMF groups, and only 60%, 57%, and 56% of cells were alive, respectively. In contrast, only 25% of the cells survived in the IF‐DOX+AMF group, which proved that IF‐DOX had an excellent combined therapeutic effect of magnetic hyperthermia and chemotherapy on tumor cells. In addition, to study the combined therapeutic effect of IF‐DOX at the cellular level more directly, the cells after different treatments were stained with calcein‐AM and PI. The fluorescent images indicated the IF‐DOX+AMF treatment led to the most obvious cell death among these groups (Figure 2d). Moreover, the apoptosis of the cells after different treatment were evaluated by flow cytometer after incubation with Annexin V‐FITC. As shown in Figure 2e, moderate cell apoptosis can be found in the DOX group, IF‐DOX group, and IF‐PBS+AMF group, and significant cell apoptosis occurred in IF‐DOX+AMF owing to the combined therapeutic effect. These results indicated that IF‐DOX implant in AMF provided highly efficient combined treatment efficacy at the cellular level.

### 2.6 Biocompatibility Assessment of IF in Vivo

**Figure 3:**
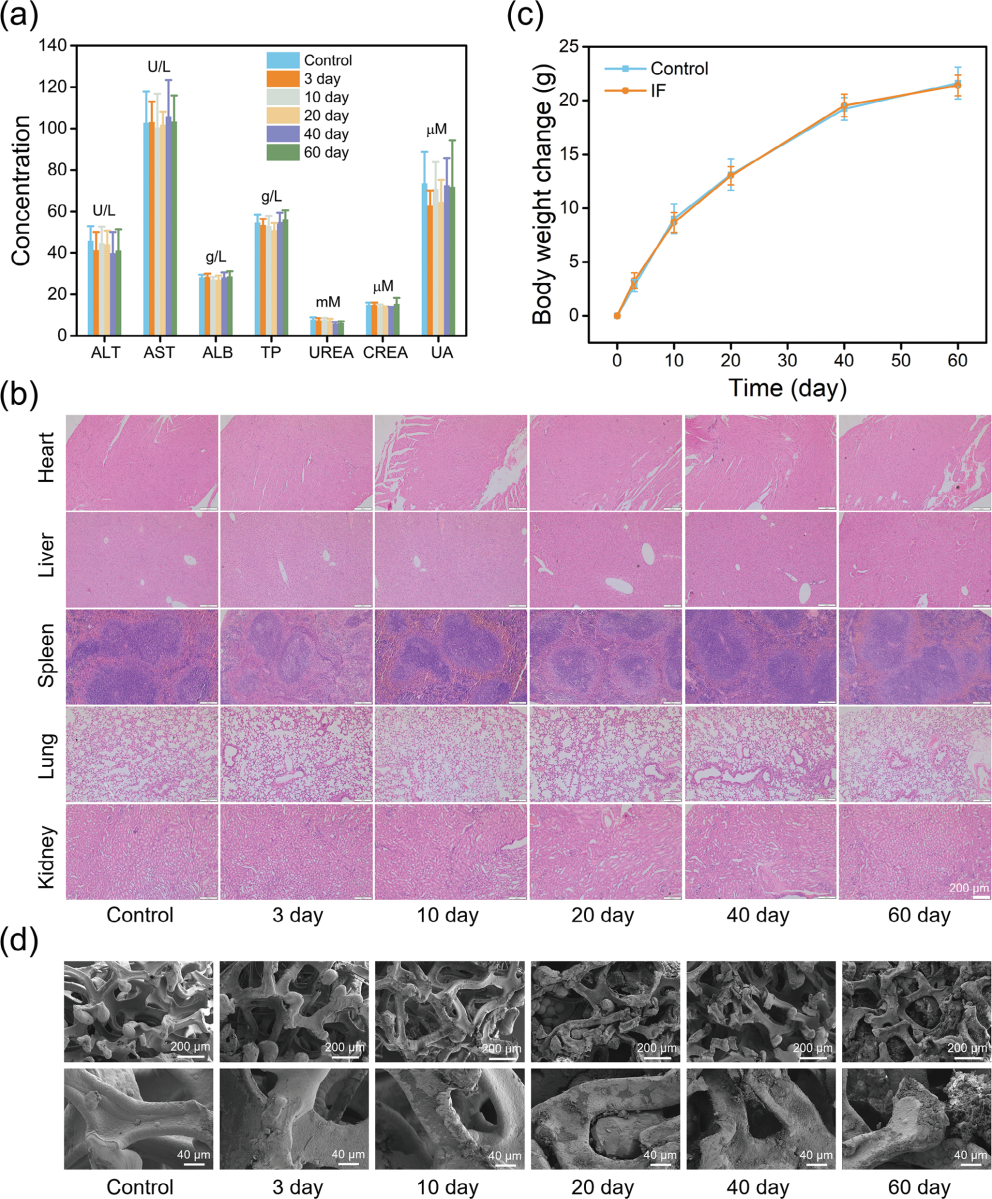
a) Serum biochemical indicators of Kunming mice after subcutaneous implantation of IF at various times. b) H&E staining results of vital organs of Kunming mice after subdermal implantation of IF at different times. c) Body weight change of Kunming mice after subdermal implantation of IF at different times. d) SEM images of IF dissected from Kunming mice after subdermal implantation at various times.

The in vivo biocompatibility of IF was evaluated on Kunming mice through blood analysis, histopathological examination, body weight monitoring, and degradation study. After being treated with the IF implant, the serum biochemical indicators of liver and kidney function of mice were all in the physiological range and had no significant difference compared to those of the blank group (**Figure** **3**a). Furthermore, the indicators of blood routine in the IF‐implantation group were all at normal levels, and there was no significant difference between the experimental group and the control group (Figure S16, Supporting Information). Inflammatory markers including tumor necrosis factor‐α (TNF‐α) and interleukin‐6 (IL‐6) in serum did not significantly increase, and the white blood cell count remained at a normal level, which indicated that implantation of IF did not cause an obvious inflammatory response (Figure S17, Supporting Information). Moreover, there was no obvious difference in tumor markers of alpha‐fetoprotein (AFP) and carcino‐embryonic antigen (CEA) in the experimental group compared with the control group, which revealed that IF did not exhibit tumorigenic effect (Figure S18, Supporting Information). In addition, the hematoxylin and eosin (H&E) staining images revealed that there was no obvious pathological change in the major organs of mice treated with IF implant (Figure 3b). Besides, there was no obvious difference in body weight change between the IF‐implantation group and the blank group within 60 days (Figure 3c). At last, on the 60th day, the IF implants in mice were collected, cleaned with water, and dried. It could be seen that there was obvious rust on the surface of the IF implant, and part of it became soft obviously (Figure 3d), which indicated IF can degrade in vivo gradually. The quantitative analysis by ICP‐OES indicated IF degraded by ≈28% after implantation in the body for 60 days (Figure S19, Supporting Information). The above results indicated the IF implant had good biocompatibility and could be used for various biological applications in vivo.

### 2.7 Computed Tomography (CT) Imaging of IF in the Tumor

**Figure 4:**
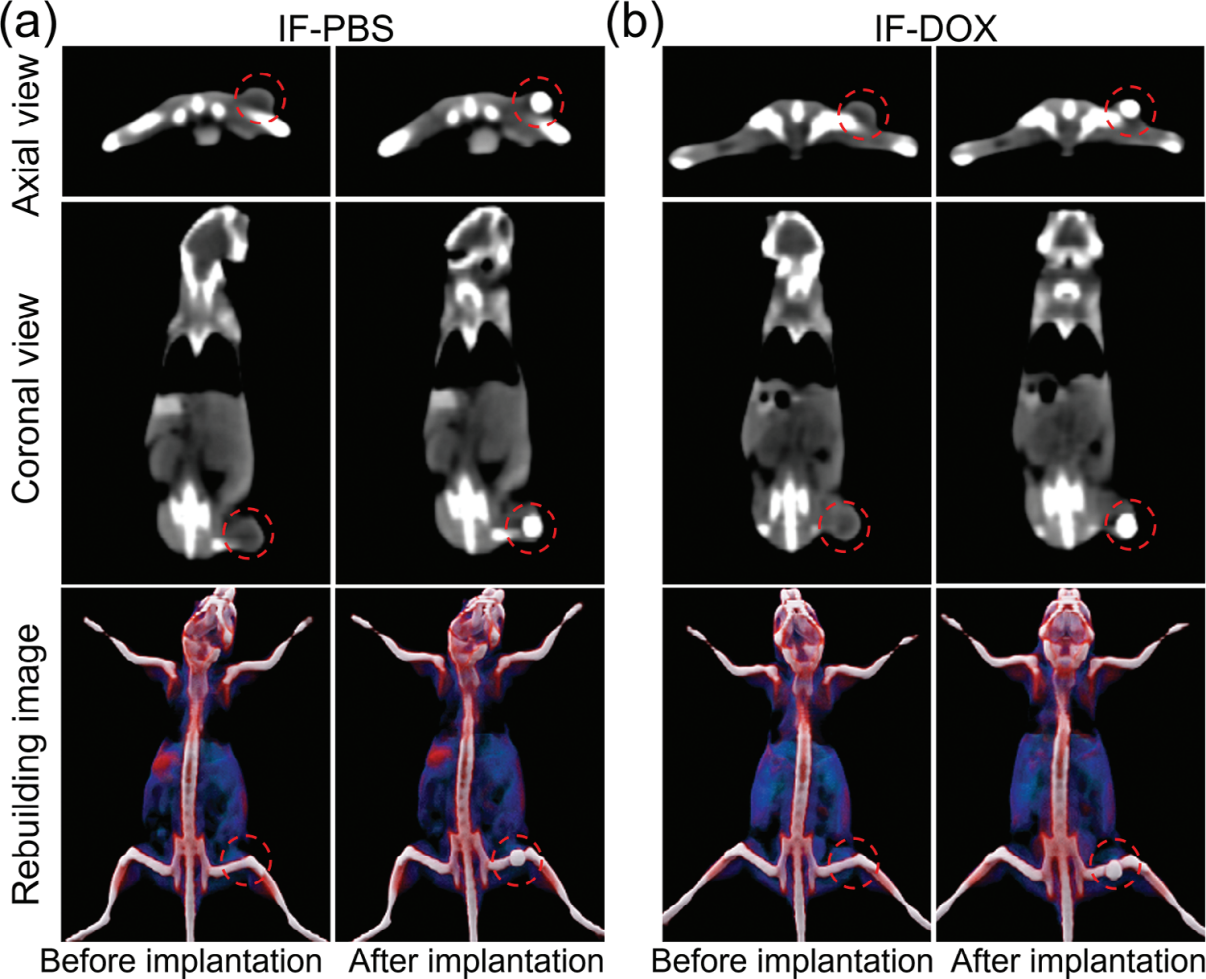
CT images of mice before and after intratumoral implantation of a) IF‐PBS and b)IF‐DOX in tumor.

Owing to the high density of iron in the IF implant, it has a strong X‐ray absorption ability, which makes it possible to ensure the successful implantation of the IF implant in the tumor by CT imaging. To evaluate the CT imaging performance of IF implants, IF‐PBS and IF‐DOX implants were implanted in the tumors of mice, and the imaging of mice was performed using a clinical CT scanner. The CT images showed that IF has good CT imaging ability and the position of IF in the tumors can be seen very clearly, which enabled CT imaging‐guided MHCT (**Figure** **4**).

### 2.8 Drug Release of IF‐Drug in Vivo

Indocyanine green (ICG) with intense near‐infrared (NIR) fluorescence was employed as a model drug to replace DOX to evaluate the drug release from IF‐drug implant in vivo. Fluorescent imaging revealed that ICG consistently maintained a high retention effect throughout the 72‐h period due to the controlled release from the implant and dense structure of tumor tissue, and then slowly diffused out of the tumor tissue as time progressed (Figure S20, Supporting Information). These results indicated the drugs in IF‐drug implants could efficiently accumulate within the tumor, facilitating an effective therapeutic outcome while reducing potential toxic side effects.

### 2.9 Combination of Magnetic Hyperthermia and Chemotherapy Using IF‐DOX for Tumors in Vivo

**Figure 5:**
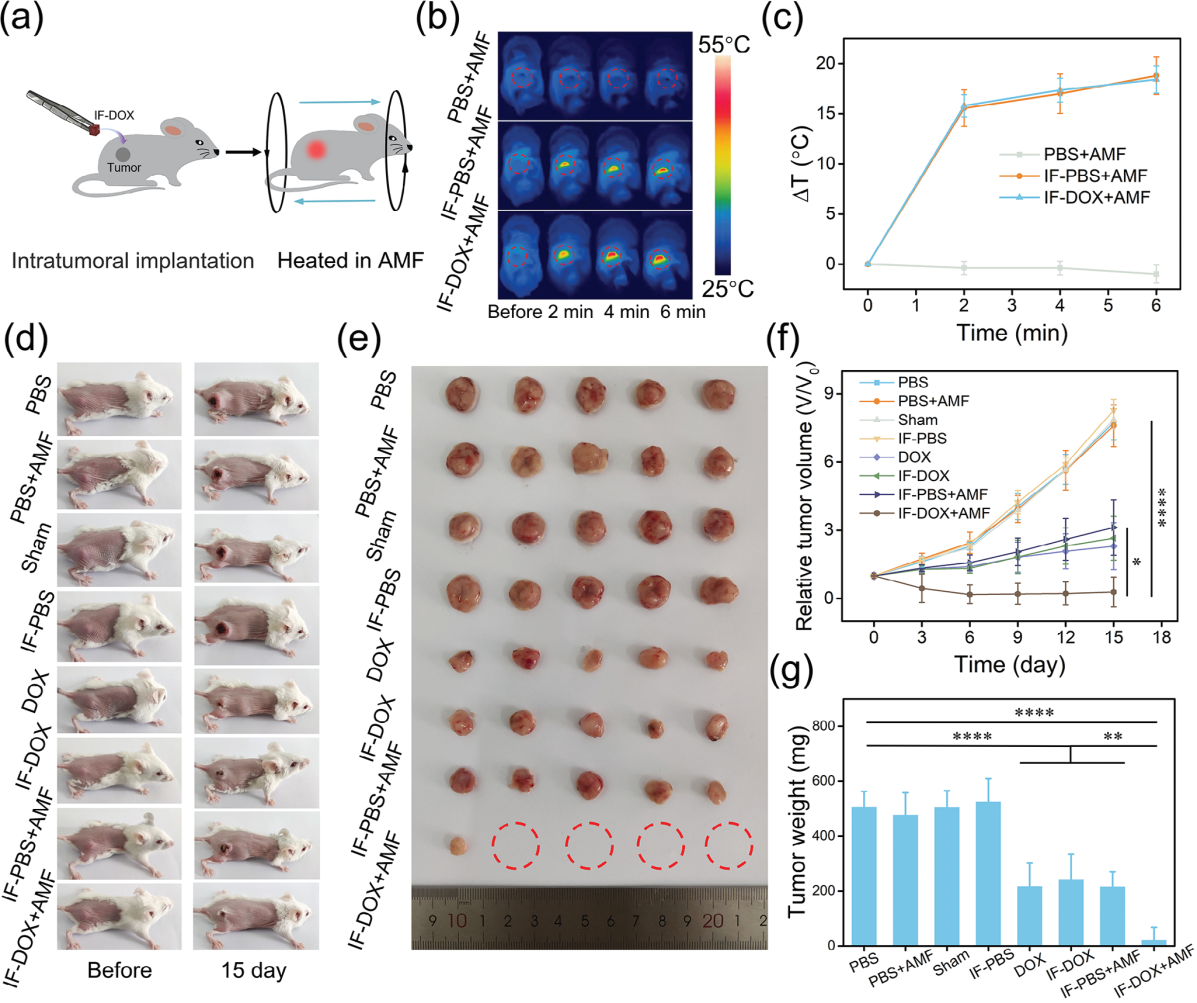
a) Schematic diagram of combined magnetothermal therapy and chemotherapy for tumors using IF‐DOX in vivo. b) Thermal images of 4T1 tumor‐bearing mice with different operations. c) Heating curves of 4T1 tumor‐bearing mice with different operations. d) Representative photos of 4T1 tumor‐bearing mice with various operations for 15 days. e) Photos of tumors dissected from mice with different treatments on the 15th day. f) Tumor growth curves of mice after various operations for 15 days. g) The weight of tumors dissected from mice after different treatments on the 15th day. Statistical analysis was determined by one‐way ANOVA; ^****^ *p* < 0.0001, ^**^ *p* < 0.01, and ^*^ *p* < 0.5.

To assess the combined therapeutic effect of IF‐DOX implant of tumors, 4T1 tumor‐bearing mice were exposed to various treatments including: i) PBS, ii) PBS+AMF, iii) Sham, iv) IF‐PBS, v) DOX, vi) IF‐DOX, vii) IF‐PBS+AMF, viii) IF‐DOX+AMF (**Figure** **5**a). The MHT in vivo

was performed under an ultralow‐power magnetic field (H_appl_·f_appl_ = 3.78 × 10^8^ A m^−1^ s^−1^), which was an order of magnitude lower than the threshold value of biosafety applications (H_appl_·f_appl_ = 5 × 10^9^ A m^−1^ s^−1^).^[^ ^13^ ^]^ As shown in Figure 5b,c, the temperature of the tumor surface increased by ≈18 °C in the IF‐PBS+AMF and IF‐DOX +AMF groups, while there was no obvious temperature elevation for tumors in PBS+AMF group exposed to the AMF, indicating the excellent magnetothermal performance of IF in vivo. Then the sizes of tumors in various groups were monitored for 15 days. Figure 5d‐g shows that there is no significant tumor inhibition effect in Sham group, PBS+AMF group, and IF‐PBS group. The DOX group, IF‐DOX group, and IF‐PBS+AMF group showed significant tumor suppression but did not result in complete tumor elimination. In contrast, the tumors in the IF‐DOX+AMF group were destroyed seriously and most of them were completely eliminated, which proved that IF‐DOX had an excellent combined therapeutic efficacy. In addition, Figure S21 (Supporting Information) shows that the tumors treated in the IF+DOX+AMF group were eliminated, which exhibited a similar therapeutic effect compared to IF‐DOX+AMF. Nevertheless, the treatment of IF+DOX+AMF will involve two operations when applied in deep tissues. More importantly, this strategy can hardly be used in tumor treatment with hydrophobic drugs. Therefore, IF‐DOX+AMF is a superior strategy for tumor treatment compared to IF+DOX+AMF. Besides, there was no significant alteration observed in the mass of IF before and after treatment, thereby suggesting that a certain degree of heating does not exert any influence on the structural integrity of IF (Figure S22, Supporting Information). The above results confirmed that the IF‐DOX implant can serve as an excellent MHCT platform for ultralow‐power magnetic hyperthermia and chemotherapy of tumors in vivo.

## 3 Conclusion

In summary, we developed 1‐min preparation of IF‐drug implant for ultralow‐power MHCT of tumors in vivo. The continuous metal structure of IF guaranteed highly efficient magnetothermal capability based on the eddy current loss mechanism, and its porous structure enabled loading drugs in an ultra‐facile manner via capillary action. In addition, IF has the merits of low cost, customizable size and shape, superior scale production capability, and good biocompatibility and biodegradability. More importantly, The IF‐drug implant is a universal platform capable of loading various hydrophilic and hydrophobic drugs by choosing appropriate and biosafe solvents. Besides, the intense X‐ray attenuation capability of IF makes it a CT imaging visible implant. We took IF‐DOX as an example to investigate the therapeutic effect of IF‐drug implants in vivo and achieved CT imaging‐guided highly efficient combination therapy of tumors under an ultralow magnetic field intensity. Our proposed IF‐drug implant solves the incompatible problem of highly efficient magnetic hyperthermia and facile drug loading and lays down a convenient and universal MHCT platform for tumor therapy in vivo.

## 4 Experimental Section

### Reagents and Materials

The ultrapure water used throughout the studies was provided by Hangzhou Wahaha Group Co., LTD. (Hangzhou, China). Doxorubicin hydrochloride (DOX), paclitaxel (PTX), cytarabine (Ara‐C), 5‐fluorouracil (5‐FU), imiquimod (R837), and manganese chloride (MnCl_2_·4H_2_O) were provided by Shanghai Aladdin Reagent Co., LTD. (Shanghai, China). Iron foam (IF) was bought from Kunshan Jiayisheng Electronics Co., LTD. (Kunshan, China). Thermochromic material was obtained from Shenzhen HUANCAIBS Co., LTD. (Shenzhen, China).

### Characterization of IF

The morphological and structural characteristics of IF were observed by scanning electron microscopy (SEM, Zeiss, Germany). The electrical conductivity of IF was characterized by a physical property measurement system (PPMS‐9, America). The drug loading ability of IF was determined by UV–vis absorption spectra by a UV‐3600 plus spectrophotometer (Shimadzu, Japan). The mass of Mn^2+^ loaded with IF was quantified by an inductively coupled plasma optical emission spectrometer (ICP‐OES, Thermo Fisher, Germany). The temperature monitoring of IF was determined by a thermal camera (Teledyne FLIR, America).

### Preparation of IF‐Drug

To investigate the kinetics of liquid loading of IF (2 × 2 × 2 mm) by the mass difference method, the mass of IFs was weighed and divided into two groups. In one group, the IFs were immersed in water by gently shaking and incubated for 30 s, 1, 3, 5, and 10 min. In the other group, the IF was exposed to ultrasonic treatment for 30 s, 1, 3, 5, and 10 min after immersing them in water. Finally, IFs with different treatments were collected and weighed, and the mass difference of IF before and after treatment was determined.

The drug loading capability of IF (2 × 2 × 2 mm) was investigated by immersing the IF (2 × 2 × 2 mm) in DOX solution with different concentrations (PBS, pH 6; 0, 1, 2, 3, 4, and 6 mg mL^−1^) for 1 min. Then the IF was taken out and added in 3 mL PBS to release the loading DOX. The relationship between the drug loading mass of IF and drug concentration was determined by UV–vis absorption spectra.

To investigate the loading capability of IF for different drugs (chemotherapeutic drugs: Ara‐C, PTX, 5‐FU, immunoregulator drugs: R837, Mn^2+^), the IFs (2 × 2 × 2 mm) were added into the drug solutions with high concentrations and gently shaken for 1 min (Ara‐C solution (H_2_O, 40 mg mL^−1^), PTX solution (ethanol, 40 mg mL^−1^), 5‐FU solution (dilute NaOH, pH 8.5, 3 mg mL^−1^), R837 solution (oleic acid, 50 °C, 20 mg mL^−1^), MnCl_2_·4H_2_O (1000 mg mL^−1^)). Then the IFs were removed from the drug solutions and placed in 3 mL of corresponding solvents, and gently shaken to release the drugs in IFs. The mass of each drug that IF can load was measured by the UV–vis absorption spectra (Ara‐C, PTX, 5‐FU, R837) and ICP‐OES (Mn^2+^).

To investigate the reproducibility of drug loading of IF, the IF (2 × 2 × 2 mm) was immersed in DOX solution (PBS, pH 6, 6 mg mL^−1^) for 1 min. Then the IF was taken out and added in 3 mL PBS to release the loading DOX. The drug loading mass of IF was determined by absorption spectra. The drug loading experiment was repeated 8 times.

### Drug Release of IF‐Drug Implant in Vitro

To investigate the drug release of IF‐drug implant in vitro, the IFs (2 × 2 × 2 mm) were immersed in DOX solution (PBS, pH 6, 6 mg mL^−1^) for 1 min, and then the IF‐DOX implant was taken out and implanted in pork tissue for different time (0, 10, 30 min, 1, 3, 6, 12, 24, and 48 h) to release the loading DOX. Thereafter the IF‐DOX implants were collected and added into 3 mL PBS to completely release residual DOX. After the solution was filtrated with a membrane filter (pore size, 0.22 µm), the amount of residual free DOX in IF was determined by UV–vis absorption spectra.

### Magnetothermal Properties of IF

To study the magnetothermal properties of IF with different sizes (2 × 2 × 2 mm, 2.5 × 2.5 × 2.5 mm, and 3 × 3 × 3 mm), IFs were put in an alternating magnetic field (AMF, frequency: 312 kHz, H = 0.46 kA m^−1^, H_appl_·f_appl_ = 1.44 × 10^8^ A m^−1^ s^−1^) and heated for 3 min, and the temperature monitoring of IF was decided by a thermal camera. In addition, the magnetothermal capability of IF (2 × 2 × 2 mm) was investigated by placing the IF (2 × 2 × 2 mm) in the AMF under different magnetic field intensities (frequency: 312 kHz, H_appl_·f_appl_ = X × 10^8^ A m^−1^ s^−1^, X = 1.44, 2.4, and 3.78) for 3 min, and the thermal camera was also used to record the temperature change. Moreover, the stability of the magnetic hyperthermia was studied by repeated heating and cooling processes in an AMF (frequency: 312 kHz, H = 1.21 kA m^−1^, H_appl_·f_appl_ = 3.78 × 10^8^ A m^−1^ s^−1^). In brief, IF (2 × 2 × 2 mm) was placed in an AMF for 3 min, and then the AMF was turned off. After the temperature of IF dropped to the initial temperature, the AMF was turned on for 3 min again. The temperature monitoring was determined by a thermal camera throughout four cycles of heating/cooling processes. To study the reproducibility of magnetothermal heating with IF, the IF (2 × 2 × 2 mm) was placed in an AMF (frequency: 312 kHz, H_appl_·f_appl_ = 1.44 × 10^8^ A m^−1^ s^−1^) for 3 min, and the temperature was monitored by a thermal camera. The heating experiment was repeated 8 times. Besides, the heating capacity of the IF was further compared with one of the best magnetic nanomaterials with a good magnetocaloric effect reported in a previous study.^[^ ^1b^ ^]^ The IF (2 × 2 × 2 mm) and cobalt ferrite (CoFe_2_O_4_) nanoparticles (powder or immersed in solution) were placed in an AMF (frequency: 312 kHz, H_appl_·f_appl_ = 1.44 × 10^8^ A m^−1^ s^−1^) for 3 min, and their temperatures were monitored by a thermal camera.

To study the tissue penetration capability of IF‐based MHT, the IF was implanted into the center of a fresh cube‐shaped pig liver (≈2 × 2 × 2 cm) coated with pork tissue with a size of ≈8 cm. The pig liver coated with pork tissue was heated in an AMF (frequency: 312 kHz, H = 2.2 kA m^−1^, H_appl_·f_appl_ = 6.86 × 10^8^ A m^−1^ s^−1^) for 6 min. Then the pig liver was cut in half to observe the color change. In the control group, 10 µL PBS was injected into the central position of pig liver tissue, and other operations were carried out the same as above. In addition, the IF (2 × 2 × 2 mm) was buried in a centrifuge tube filled with thermochromic material (light pink to amaranth at >60 °C) and implanted in pork tissue with the sizes of 8 cm. Then the pork tissue was placed in an AMF for 6 min (frequency: 312 kHz, H = 2.2 kA m^−1^, H_appl_·f_appl_ = 6.86 × 10^8^ A m^−1^ s^−1^).

To study the magnetothermal ability of IF before and after loading drugs, IFs (2 × 2 × 2 mm) immersed in PBS and DOX (6 mg mL^−1^) solution were placed in AMF (frequency: 312 kHz, H = 1.21 kA m^−1^, H_appl_·f_appl_ = 3.78 × 10^8^ A m^−1^ s^−1^) for 3 min, and the temperature monitoring was determined by the thermal camera.

### Degradation of IF in Vitro

The degradation of IF in vitro was investigated by the mass difference method. The IF was weighed and added to 1 mL of water and saline at room temperature for different time durations (0, 3, 10, 20, 40, and 60 days). Then the IF was taken out, washed with water, dried, and weighed, and the degradation degree of IF in various solutions was determined.

### Cytotoxicity of IF

4T1 cells were used to study the cytotoxicity of IF by standard MTT assay. 4T1 cells were seeded into cell culture plates and cultured in a cell incubator for 24 h. The old medium was thrown, and the adherent cells were washed using PBS. Then 180 µL new medium and the IF (2 × 2 × 2 mm) were added, and a small magnet was placed on the outer side of the 96‐well plates to make IF stick to the inner side of the wells to reduce the damage to cells caused by mechanical compression. After incubating for different time durations (6, 12, and 24 h), the IF, small magnet, and old medium were removed and the treated cells were washed using PBS. Then 200 µL new medium and 10 µL thiazole blue (5 mg mL^−1^) were added and co‐incubated for 4 h. At last, the supernatants in the cells were thrown away and 120 µL Dimethylsulfoxide (DMSO) was added to dissolve formazan. The cell viabilities were determined by measuring the absorbance of the solution (490 nm) in each well using a microplate reader (BioTek, USA).

### In Vitro Combination Therapy Using IF‐DOX

To investigate the combination therapy effect of IF‐DOX in vitro, 4T1 cells were cultured on cell culture plates in a cell incubator for 24 h. After removing old media and washing using PBS, the 4T1 cells were subjected to different treatments: i) PBS group: 180 µL new medium and 10 µL PBS; ii) PBS+AMF group: 180 µL new medium and 10 µL PBS and putting in AMF for 7 min; iii) IF‐PBS group: 180 µL new medium and one IF‐PBS; iv) DOX group: 180 µL new medium and 10 µL DOX (3.8 µg); v) IF‐DOX group: 180 µL new medium and one IF‐DOX (DOX: 3.8 µg); vi) IF‐PBS+AMF group: 180 µL new medium and one IF‐PBS and putting in AMF for 7 min; vii) IF‐DOX+AMF group: 180 µL new medium and one IF‐DOX (DOX: 3.8 µg) and putting in an AMF for 7 min. The parameters of AMF used in cellular therapy were as follows: frequency = 362 kHz, H = 1.01 kA m^−1^, H_appl_·f_appl_ = 3.66 × 10^8^ A m^−1^ s^−1^. It is worth noting that to eliminate mechanical compression damage caused by the weight of IF, small magnets were placed on the outer side of the 96‐well plates after heating in the AMF to make the IF attach to the inner side wall of the wells. The cells were placed in a cell incubator and incubated for 24 h, and then the small magnets, IF, and old medium were removed. After washing with PBS, 200 µL of new medium and 10 µL of thiazole blue (5 mg mL^−1^) were added and co‐incubated for 4 h. At last, the supernatants in the cells were thrown away and 120 µL DMSO was added to dissolve the generating formazan. The cell viabilities were determined by measuring the absorbance of the solution (490 nm) in each well using a microplate reader. In addition, to study the combined killing effect of the IF‐DOX on cells more directly, the cells with different treatments were stained using calcein‐AM and PI and observed under a fluorescence microscope to analyze the survival status of the treated cells. Moreover, to further analyze the apoptosis status of cells in each treatment group, the cells were incubated with Annexin V‐FITC and determined by flow cytometry.

### Biocompatibility of IF in Vivo

All animal‐associated experiments were conducted in accordance with the guidelines of the Animal Care and Use Committee of Tianjin Medical University and obtained approval from the Animal Care and Use Committee of Tianjin Medical University (SYXK: 2019‐0004). Kunming mice (≈20 g, SPF Biotechnology Co., Ltd., Beijing, China) were used to evaluate the biocompatibility of IF in vivo by blood analysis, histopathological examination, body weight monitoring, and degradation study. Blood and serum were collected at different time durations (3, 10, 20, 40, and 60 days) after subcutaneous transplantation of IF for blood analysis. The analyzed indicators included liver function indexes (Alanine aminotransferase (ALT), Aspartate aminotransferase (AST), Albumin (ALB) and total protein (TP)), kidney function indicators (serum creatinine (CREA), urea (Urea) and Uric Acid (UA)), inflammatory markers (tumor necrosis factor‐α (TNF‐α) and interleukin‐6 (IL‐6)), tumor markers (alpha fetoprotein (AFP) and carcino‐embryonic antigen (CEA)) and blood routine indicators (leukocyte count (WBC), lymphocyte count (Lymph#), monocyte count (Mon#), neutrophil number (Gran#), lymphocyte percentage (Lymph%), monocyte percentage (Mon%), neutrophilic granulocyte percentage (Gran%), erythrocyte count (RBC), hemoglobin (HGB), hematocrit (HCT), mean corpuscular volume (MCV), mean erythrocyte hemoglobin content (MCH), mean corpuscular‐hemoglobin concentration (MCHC), red blood cell distribution width (RDW), platelet count (PLT), mean platelet volume (MPV), platelet distribution width (PDW), plateletcrit (PCT)). At the same time, major organs (heart, liver, spleen, lung, and kidney) of Kunming mice in different groups were dissected and carried out with Hematoxylin and Eosin (H&E) staining. In addition, the body weight change of Kunming mice in IF implanted group and control group was monitored within 60 days. Besides, IFs implanted under the skin of mice at different time points were dissected, washed, dried, and analyzed by ICP‐OES and SEM.

### CT Imaging of IF in Tumors

4T1 tumor‐bearing Balb/c mice (≈20 g, SPF Biotechnology Co., Ltd., Beijing, China) were used to evaluate the CT imaging capability of IF. Balb/c mice were subcutaneously inoculated with tumor cells on the right back, and in vivo experiments were carried out when the diameter of the tumor grew to ≈5–7 mm. The CT imaging was performed using a CT scanner in the clinic (Erlangen, Germany) after IF‐PBS and IF‐DOX were transplanted into the tumor of Balb/c mice. The tube voltage was 80 kV, and the volume render (VR) method was adopted to reconstruct 3D images based on CT images.

### Drug Release of IF‐Drug Implant in Vivo

To investigate the drug release behavior in vivo, ICG with intense near‐infrared (NIR) fluorescence was chosen as a model drug to replace DOX to explore the drug release from IF‐drug implant in vivo. After implantation of IF (2 × 2 × 2 mm) or IF‐ICG (ICG: 0.08 µg) in the tumor, the mice were imaged by a small animal fluorescent imaging system at different time points.

### Combined Magnetic Hyperthermia and Chemotherapy of Tumors Using IF‐DOX in Vivo

4T1 tumor‐bearing mice were used to study the combined therapeutic efficacy of IF‐DOX in vivo. The mice were randomly divided into 8 groups for different treatments (*n* = 5): i) PBS group: intratumoral injection of 10 µL PBS; ii) PBS+AMF group: intratumoral injection of 10 µL PBS and heated in AMF for 6 min; iii) Sham group; iv) IF‐PBS group: implantation of IF‐PBS in tumor; v) DOX group: intratumoral injection of 10 µL DOX (48 µg); vi) IF‐DOX group: implantation of IF‐DOX (DOX: 48 µg) in tumor; vii) IF‐PBS+AMF group: implantation of IF‐PBS in the tumor, and heated in an AMF for 6 min; viii) IF‐DOX+AMF group: implantation of IF‐DOX (DOX: 48 µg) in the tumor, and heated in an AMF for 6 min. The parameters of AMF used in vivo therapy were as follows: frequency = 312 kHz, H = 1.21 kA m^−1^, H_appl_·f_appl_ = 3.78 × 10^8^ A m^−1^ s^−1^. Additionally, as the preparation of IF‐DOX is extremely simple, a ready‐to‐use strategy was adopted for combined therapy of tumors in vivo. From the preparation of the implant, transplantation into the live tumor, and wound closure, to the initiation of magnetothermal therapy, this entire process took less than 5 min. Besides, after implantation of IF in the tumor and wound closure, the mice were instantly placed in an AMF for magnetothermal heating. All mice were carried out with the same process.

The temperature monitoring of mice during the heating process in AMF was using a thermal camera. The mice in each group were monitored for 15 days and tumor length and width were determined by a digital caliper every 3 days. Tumor volume was defined as volume = length × width^2^/2. In addition, the IF was initially weighed prior to treatment, subsequently extracted, cleaned, dried, and then reweighed for the purpose of comparing the mass changes before and after treatment.

## Conflict of Interest

The authors declare no conflict of interest.

## Supporting information

Supporting Information

## Data Availability

The data that support the findings of this study are available from the corresponding author upon reasonable request.
