# Supplementary material for: One‐Minute Preparation of Iron Foam‐Drug Implant for Ultralow‐Power Magnetic Hyperthermia‐Based Combination Therapy of Tumors in Vivo
Source: Adv Sci (Weinh). 2024 Jan 2;11(11):2307823. doi: 10.1002/advs.202307823 (PMC10953590; doi:10.1002/advs.202307823)
Supplement: Supplementary file 1 — Supporting Information [file ADVS-11-2307823-s001.pdf]

ADVANCED SCIENCE
Open Access

Supporting Information

for *Adv. Sci.*, DOI 10.1002/advs.202307823

One-Minute Preparation of Iron Foam-Drug Implant for Ultralow-Power Magnetic Hyperthermia-Based Combination Therapy of Tumors in Vivo

*Guangchao Xie, Bingjie Li, Xuejun Zhang, Jiaojiao Yu and Shao-Kai Sun**

Supporting Information

One minute preparation of iron foam-drug implant for ultralow-power magnetic hyperthermia-based combination therapy of tumors in vivo

*Guangchao Xie, Bingjie Li, Xuejun Zhang, Jiaojiao Yu, and Shao-Kai Sun**

G. Xie
Department of Diagnostic and Therapeutic Ultrasonography, Tianjin Medical University Cancer Institute and Hospital, National Clinical Research Center of Cancer, Key Laboratory of Cancer Prevention and Therapy, Tianjin 300060, China

G. Xie, X. Zhang, J. Yu, S. Sun
School of Medical Imaging, Tianjin Medical University, Tianjin 300203, China
E-mail: shaokaisun@tmu.edu.cn

B. Li
Department of Radiology and Tianjin Key Laboratory of Functional Imaging, Tianjin Medical University General Hospital, Tianjin, 300052, China

Keywords: Iron foam, Combination treatment, Low-power magnetic hyperthermia, Facile drug loading

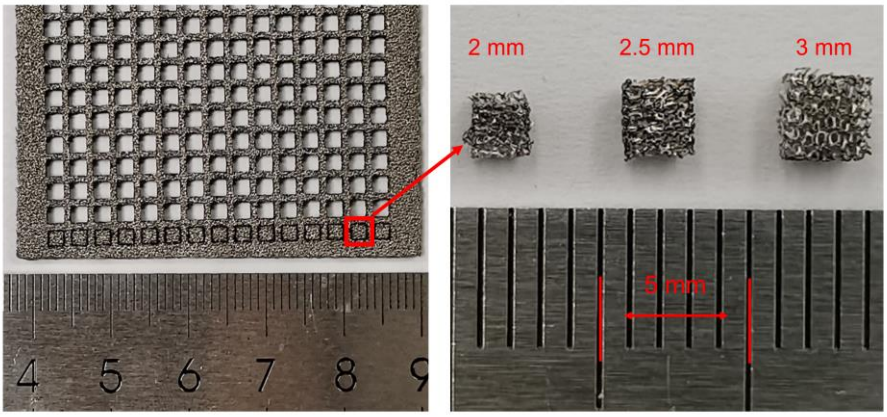

Figure S1. Photos of IF cubes with different sizes (2×2×2 mm, 2.5×2.5×2.5 mm, 3×3×3 mm).

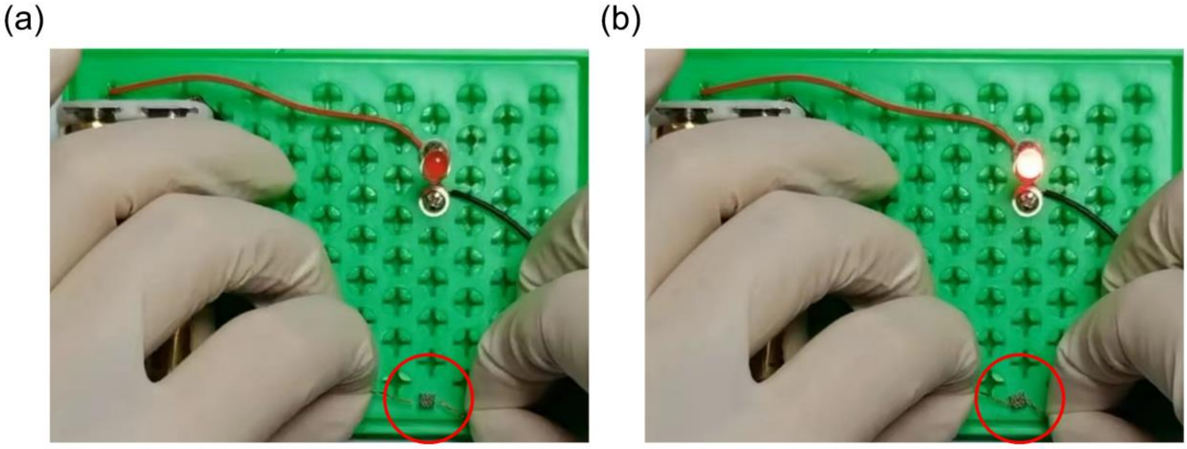

Figure S2. (a) Photos of experiment on IF conductivity (turn off). (b) Photos of experiment on IF conductivity (turn on).

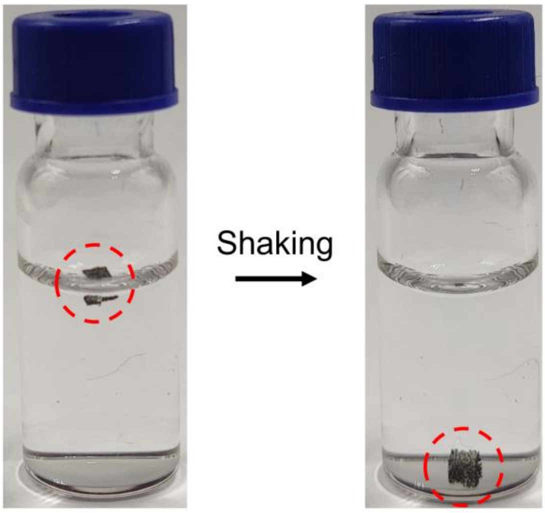

Figure S3. Photos of IF (2×2×2 mm) in water before and after shaking.

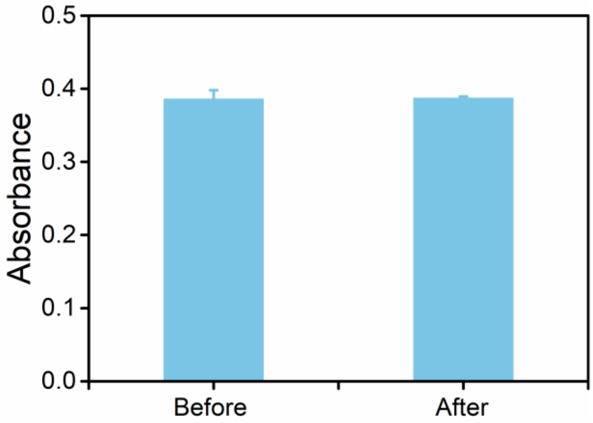

Figure S4. Absorbance of drug solution before and after IF loading.

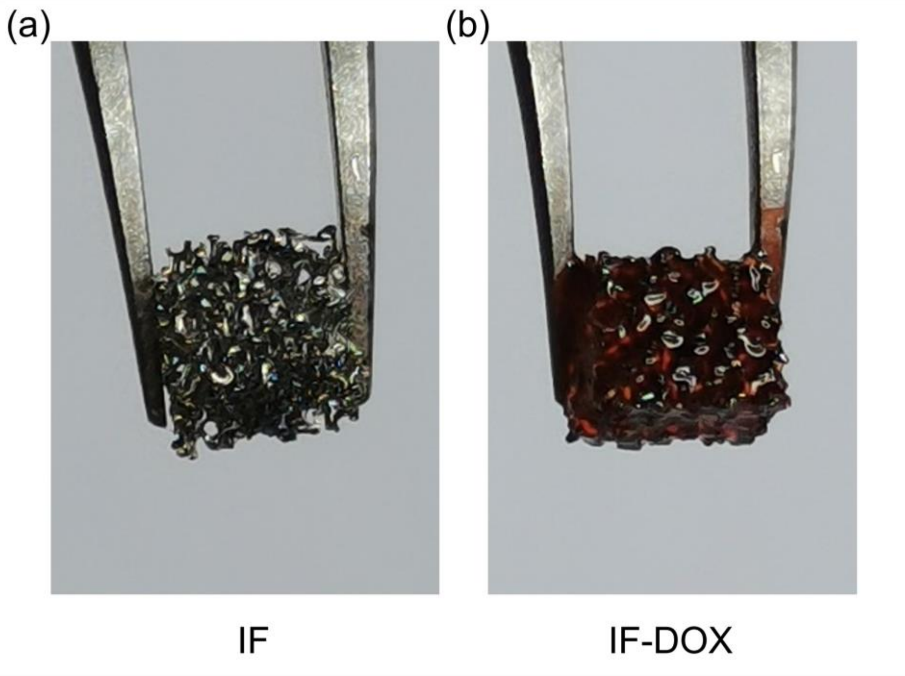

Figure S5. Photos of IF and IF-DOX.

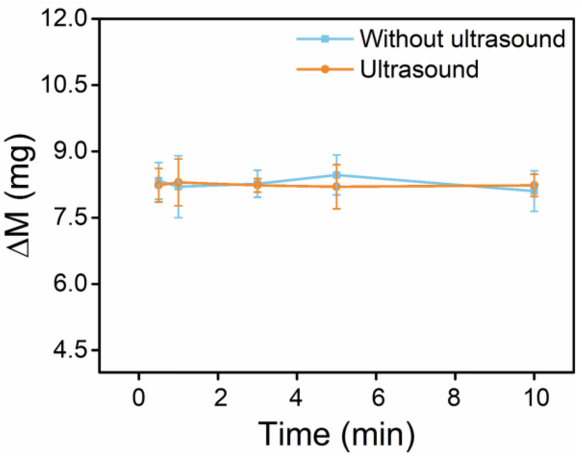

Figure S6. The mass of water loaded by one IF for different time with or without ultrasound (The actual mass of the loaded liquid was slightly greater than the theoretical value calculated from the porosity because the surface of the IF absorbed a small amount of liquid).

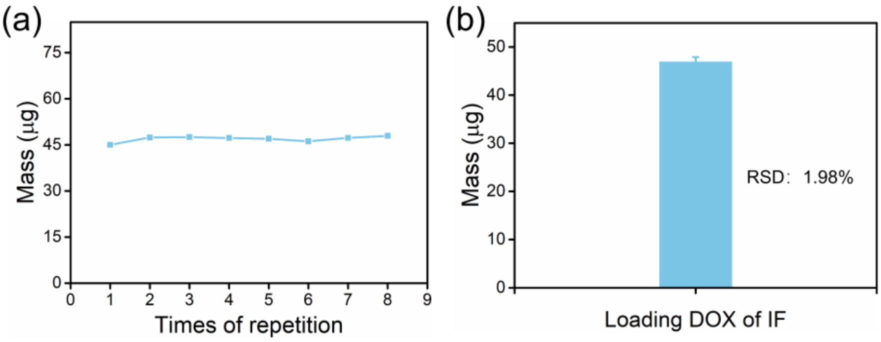

Figure S7. Reproducibility experiment of IF loaded with DOX. (a) Mass of DOX loaded by IF with 8 repetitions. (b) RSD of reproducibility experiment of IF loaded with DOX.

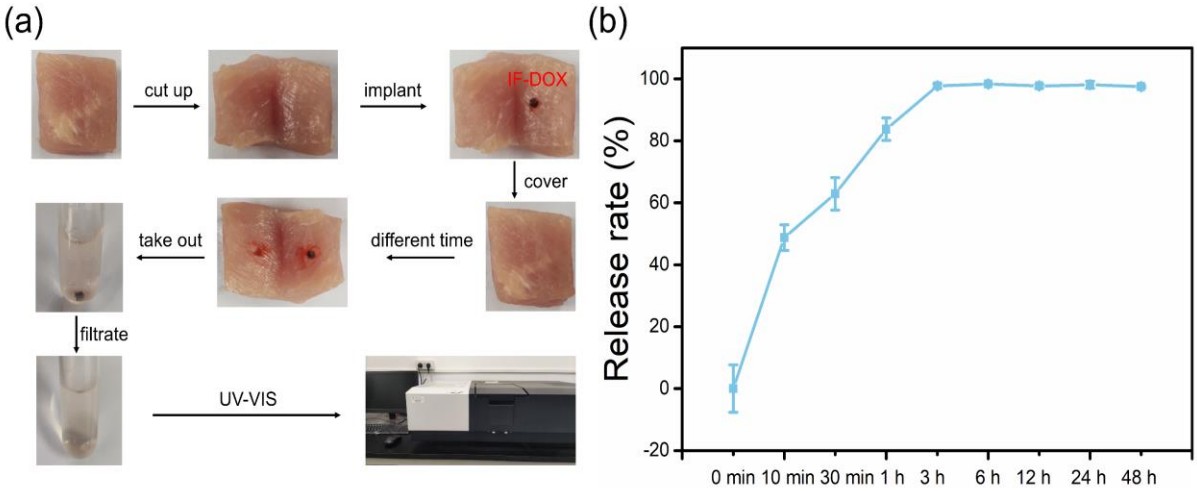

Figure S8. (a) Schematic diagram for measurement of the release of IF-DOX implant in vitro. (b) The release rate of IF-DOX implant in vitro.

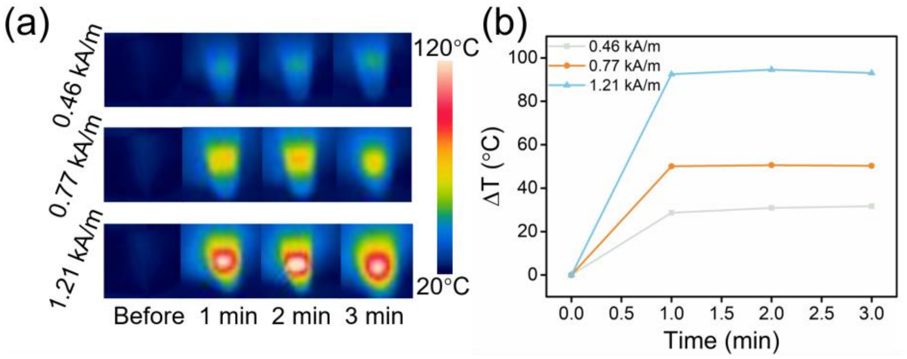

Figure S9. (a) Thermal images of IF in AMF under various magnetic field intensities. (b) Heating curves of IF in AMF under various magnetic field intensities.

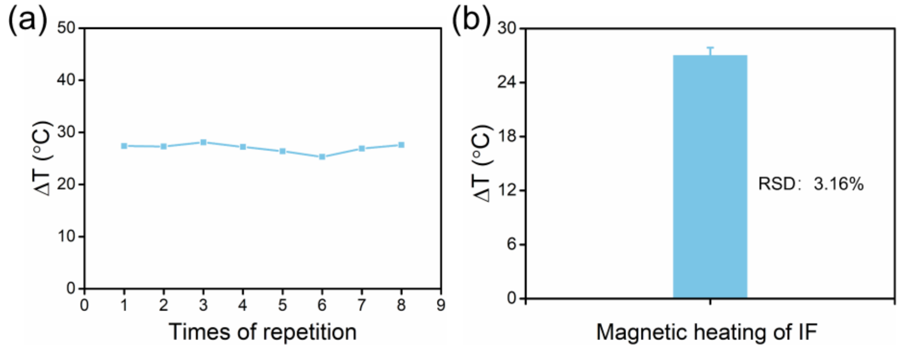

Figure S10. Reproducibility experiment of magnetic heating of IF in an AMF. (a) Temperature change of IF in an AMF with 8 repetitions. (b) RSD of reproducibility experiment of magnetic heating of IF in an AMF.

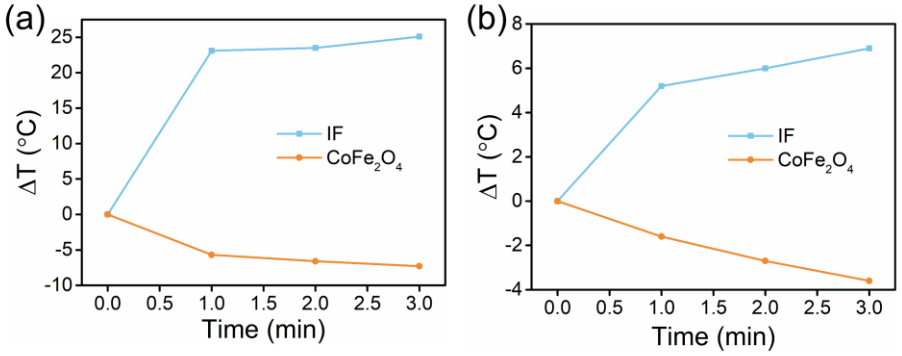

Figure S11. (a) Heating curves of IF and $CoFe_2O_4$ nanoparticle in an AMF (powder: 4.7 mg). (b) Heating curves of IF and $CoFe_2O_4$ nanoparticle in an AMF (immersed in H_2O: 16.8 mg/mL).

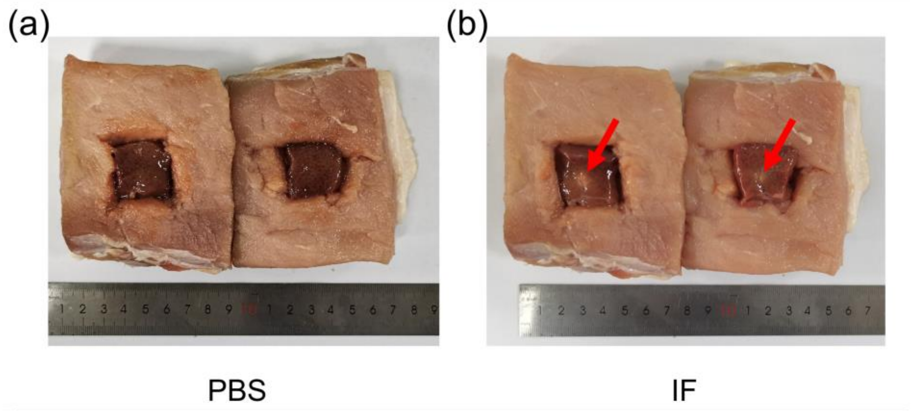

Figure S12. Photos of dissected pig liver coated with pork tissue after treated with PBS or IF in an AMF.

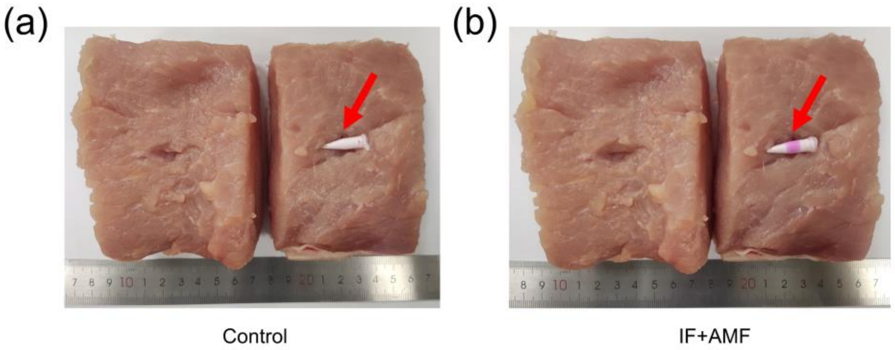

Figure S13. Photos of a centrifuge tube filled with thermochromic material (light pink to amaranth at >60°C) and coated in pork tissue after treated with IF or not in an AMF.

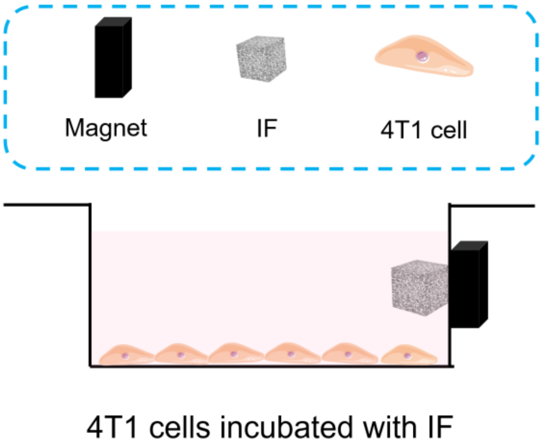

Figure S14. Schematic diagram of 4T1 cells incubated with IF at the cellular level.

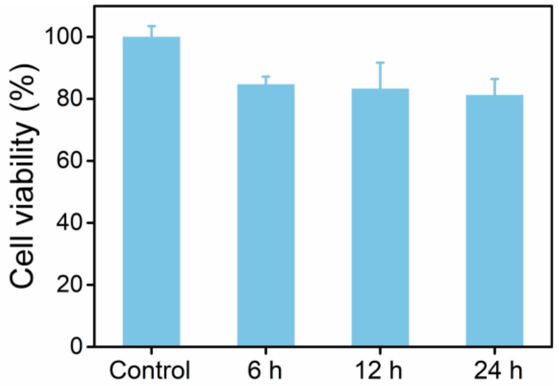

Figure S15. The viabilities of 4T1 cells after treating with IF for various time.

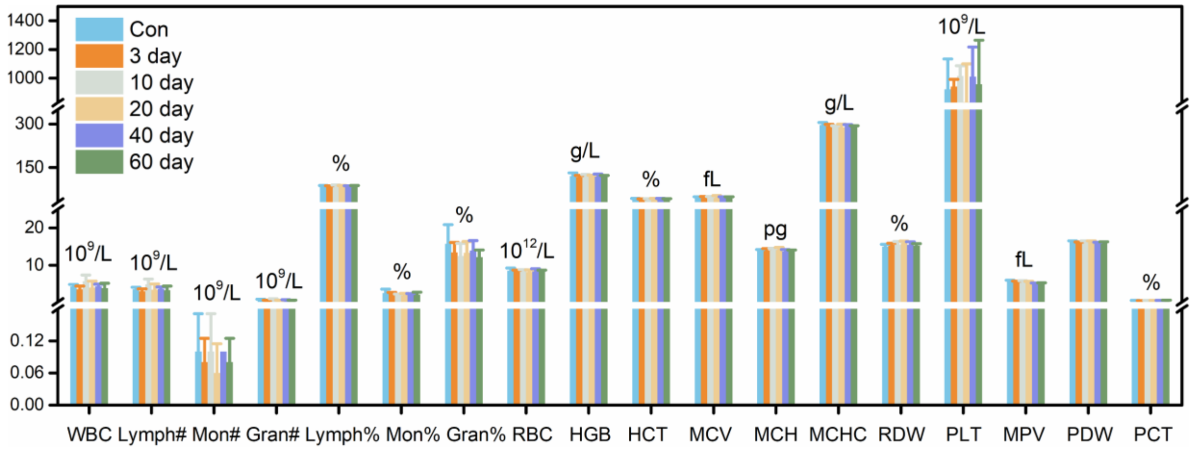

Figure S16. The blood routine results of mice after implantation of IF for different time (WBC: leukocyte count; Lymph#: lymphocyte count; Mon#: monocyte count; Gran#: neutrophil number; Lymph%: lymphocyte percentage; Mon%: monocyte percentage; Gran%: neutrophilic granulocyte percentage; RBC: erythrocyte count; HGB: hemoglobin; HCT: haematocrit; MCV: mean corpuscular volume; MCH: mean erythrocyte hemoglobin content; MCHC: mean corpuscular-hemoglobin concentration; RDW: red blood cell distribution width; PLT: platelet count; MPV: mean platelet volume; PDW: platelet distribution width; PCT: plateletcrit).

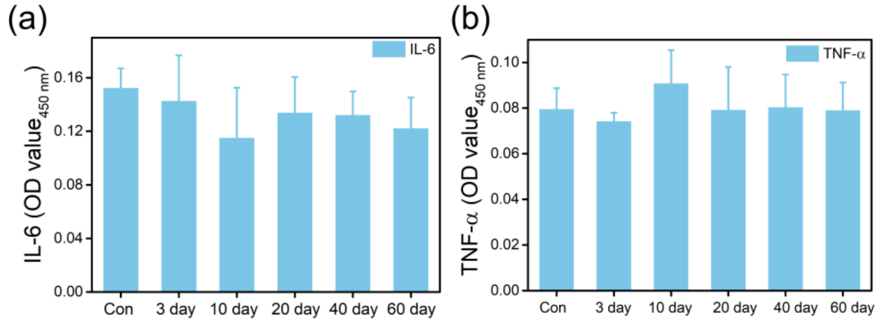

Figure S17. The Elisa results of IL-6 and TNF-α in serum from mice after implantation of IF for different time.

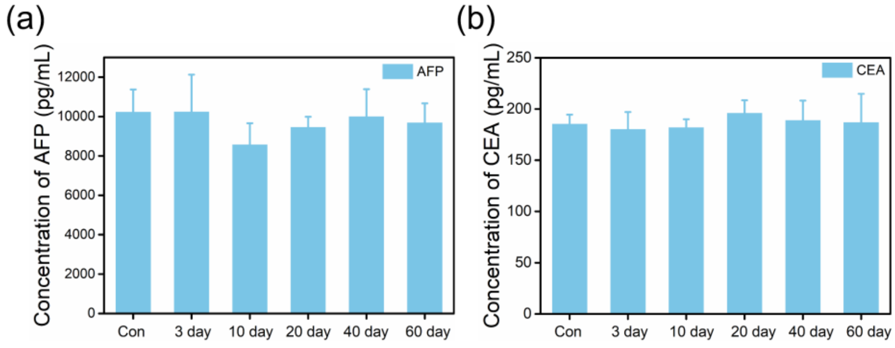

Figure S18. The Elisa results of AFP and CEA in serum from mice after implantation of IF for different time.

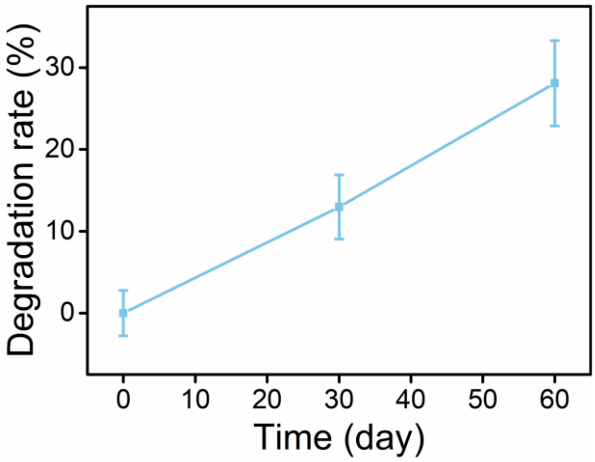

Figure S19. The degradation rate of IF after implantation in mice for various time.

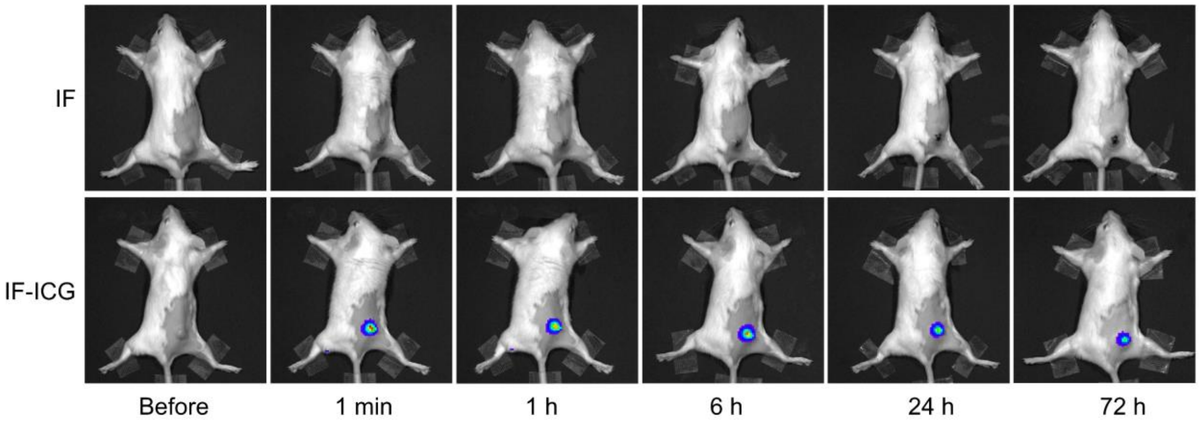

Figure S20. The fluorescent images of mice after implantation of IF (2×2×2 mm) or IF-ICG (ICG: 0.08 μg) in tumor for different time.

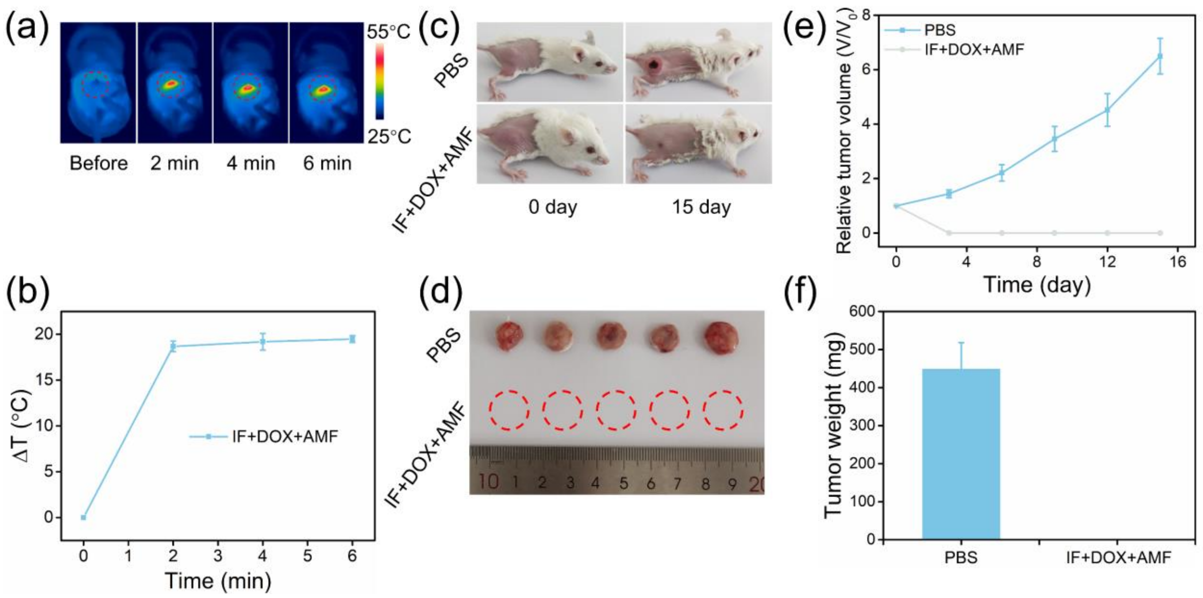

Figure S21. (a) Thermal images of 4T1 tumor-bearing mice with different operations. (b) Heating curves of 4T1 tumor-bearing mice with different operations. (c) Representative photos of 4T1 tumor-bearing mice with various operations for 15 day. (d) Photos of tumors dissected from mice with different treatments on the 15th day. (e) Tumor growth curves of mice after various operations for 15 day. (f) The weight of tumors dissected from mice after different treatments on the 15th day.

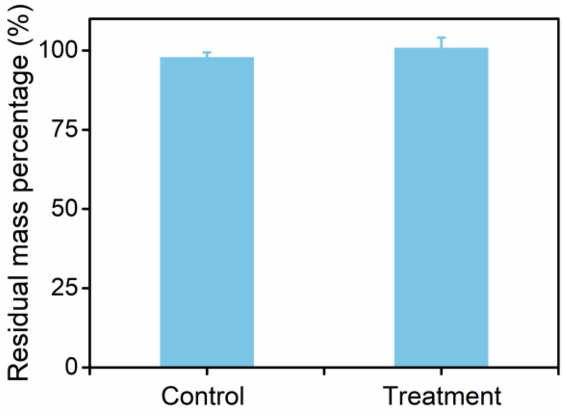

Figure S22. Residual mass percentage of IF after tumor treatment by IF-DOX implant.
